# Trends of cancer incidence and mortality in Cali, Colombia. 50 years experience

**Published:** 2012-12-30

**Authors:** Luis Eduardo Bravo, Tito Collazos***, Paola Collazos, Luz Stella García, Pelayo Correa

**Affiliations:** a Cancer Registry of Cali, Departament of Pathology. Universidad del Valle, Cali, Colombia E-mail: bravo.luiseduardo@gmail.com; *** Deceased Former administrator of the Registro Poblacional de Cancer de Cali; b Division of Gastroenterology, Department of Medicine, Vanderbilt University Medical Center, Nashville, TN, USA. E-mail: pelayo.correa@vanderbilt.edu

**Keywords:** Cancer, epidemiology, Cali, Colombia, cancer trends

## Abstract

**Purpose:**

The Population-based Cancer Registry of Cali aims to report all new cases in permanent residents within the limits of the city of Cali. Time trends of cancer incidence and mortality are described. The registry has been in continuous operation for 50 years.

**Methods:**

Cancer cases reports are obtained actively by visiting all sources of information: hospitals, pathology departments, hematology laboratories, radiotherapy centers, government offices where death certificates are processed and physician's offices. It is estimated that the reporting is at least 95% complete.

**Results:**

Drastic decreases are documented in rates for tumors causally related to infectious agents, especially cancers of the uterine cervix and the stomach. Gradual increases are documented in rates of tumors linked to affluence and the metabolic syndrome, especially cancers of the colon and the female breast. An unexpected increase in the incidence of papillary carcinoma of the thyroid gland in women is reported. Tobacco-related cancers, especially cancer of the lung, showed marked increase in incidence rates around 1970, apparently the beginning of an epidemic similar to the one reported in Western societies. But the increase in incidence stopped around 1980, resulting from a strong anti-smoking campaign launched in Colombia in the 1970s.

**Conclusions:**

The findings have influenced prevention strategies implemented by public health authorities, specially the establishment of a city-wide program to prevent cervix cancer via widespread use of vaginal cytology and anti-smoking campaigns. Also, new population-based cancer registries have been established in other Colombian cities as well as in Ecuador.

## Abstract (es)

**Propósito:**

El objetivo del Registro poblacional de cáncer (RPCC) de Cali, es registrar todos los casos nuevos en residentes permanentes dentro de los límites de Cali. Se describen las tendencias de la incidencia y mortalidad. El RPCC ha estado en operación continua por 50 años.

**Metodología:**

Los casos registrados son obtenidos mediante búsqueda activa en las fuentes de información como: hospitales, laboratorios de Patología y Hematología, centros de Radioterapia, oficina estatales en las que se procesan los certificados de defunción y consultorios de médicos privados. Se estima que el proceso de registro es completo en un 95%.

**Resultados:**

Se describe una disminución drástica en la incidencia de tumores relacionados con agentes infecciosos, especialmente los casos de cáncer de cuello uterino y estómago. Se documenta incremento gradual en las tasas de incidencia de los tumores asociados con la afluencia y el síndrome metabólico, especialmente el cáncer de colon y mama en la mujer. Se advierte un aumento inesperado de la incidencia de carcinoma papilar en la glándula tiroides en mujeres. Los tipos de cáncer relacionados con el consumo de tabaco, especialmente el de pulmón, mostraron un aumento de la incidencia alrededor de 1970, aparentemente el comienzo de una epidemia similar a la reportada en sociedades occidentales. Pero el incremento de la incidencia se detuvo en 1980, resultado de una fuerte campaña anti-tabaco liderada en Colombia en los setenta.

**Conclusiones:**

Los resultados han influido en las estrategias de prevención aplicadas por las autoridades de salud pública, en especial el establecimiento de un programa para toda la ciudad para prevenir el cáncer de cuello uterino a través del uso generalizado de la citología vaginal y las campañas contra el tabaquismo. Además, se han establecido nuevos registros poblacionales de cáncer en otras ciudades de Colombia y en Ecuador.

## Introduction

Reliable data on time trends in cancer incidence and mortality are for the most part unavailable in Latin America. The main reason is the scarcity of population-based cancer registries in continuous operation for prolonged periods of time. The Population-based Cancer Registry of Cali, Colombia (Registro Poblacional de Cancer de Cali, RPCC) started 50 years ago as a 2 year incidence survey. The objective was to register all new cancer cases diagnosed in the city during 1962 and 1963. The survey was a joint project of the departments of Pathology and Preventive Medicine of Universidad del Valle School of Medicine. A team of medical students were especially trained to review and abstract the records of 16 medical institutions (1504 hospital beds) and 483 practicing physicians attending patients in whom cancer could be diagnosed and/or treated. The survey emphasized registering only new cases in patients who were permanent residents of the city, living within bounds clearly demarcated by the municipality.

At the time there were no data on cancer incidence in Colombia. Data on mortality were unreliable; some death certificates were written by non-medical personnel and were not accurate about the cause of death. Some data were available on the relative frequency of cancer in hospitals, but were biased by the selective admission policies of the institution. One study reported relative frequency for all cases diagnosed in the department (state) of Antioquia, based on all histopathology reports for the area ^1^ ^,^ ^2^. The Cali survey therefore intended to fill the vacuum on information about cancer frequency in Colombia. The results of the survey were published in national and international medical journals ^3^ ^-^ ^5^. They revealed high rates for cancer of all sites combined, driven in men by gastric cancer and in women by carcinoma of the uterine cervix. The latter was of epidemic proportions. Low rates of colon and lung cancer were reported. These findings stimulated further research and were instrumental in launching a public health campaign to control cervical cancer, especially by setting up multiple centers for collection of vaginal cytology specimens, processed in a central laboratory under the direction of a specialized pathologist^6^. In the following years, the rates of carcinoma in situ increased considerably and coincided with a decrease in invasive cervical carcinoma^5^. Because of the success of the survey, the decision was made to continue the Registry as a permanent endeavor, based in the department of Pathology, mostly with resources provided by the University. Systems for capturing the information in a permanent basis were set up in the main institutions, complemented by the historically proven successful collaboration of medical students.

### The population

**Figure 1:**
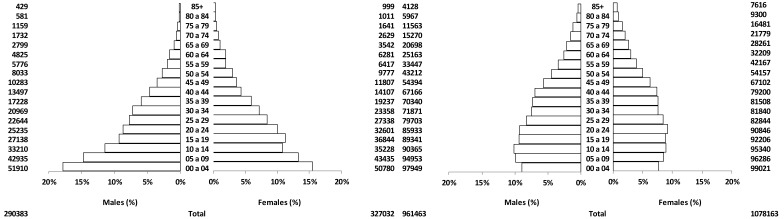
Cali, Colombia. Population structure by age and sex.

At that time of the establishment of the Registry the city had 578,440 inhabitants; approximately 64% of them were immigrants, mostly from other regions in Colombia, reflecting economic and political pressures in some rural areas, driving internal migration. A national census was carried out on July 15, 1964. It contained detailed information on the place of birth of each resident. This allowed the construction of population pyramids for subjects born in the city as well as for the main immigrant groups. This demographic information allowed the calculation of cancer incidence specific rates for each group of residents^7^. Population pyramids were drawn for natives and the main immigrant groups. The population of Cali in 1964 displayed a pattern closer to the "African Model" with wide base and narrow apex (Fig. 1). It reflects the influence of immigrant s from rural southern populations (Cauca and Nariño) with a typical African-type demographic structure and immigration from urban populations with a pattern closer to the "European" model (Bogota and Antioquia)^7^.The population of Cali in 1964 changed into a more "European" pattern in 2005, when the population had grown to 2,309,626. The demographic structure then is seen in Fig. 1. The aging index (population 65 + years divided by children under 15 years) is approximately 0.041 in 1964 and 0.2472 in 2005. Therefore, the 1964 population was considerably "younger" than that of 2005. These data show a remarkable transformation of the population of Cali in approximately 40 years.

## Materials and Methods

In 2010 the Registry covered the population of the urban area of Cali with 119 square kilometers. Cali is the capital of the Department (state) of Valle del Cauca, one of the 32 departments in which the country is divided politically. Its demographic distribution is shown in Fig. 1. Most of the inhabitants are mestizos (ancestral admixture of Amerindians and Caucasian). More than one half are immigrants, mostly from other parts of Colombia. Cali is situated 1,000 meters above sea level at latitude 3-27 N and longitude 7-31 W. The average temperature is 24 degrees Celsius.

The RPCC is a program of the Department of Pathology of Universidad del Valle. Cancer cases reports are obtained actively by visiting all sources of information. These sources include hospitals, pathology departments, hematology laboratories, radiotherapy centers, government offices where death certificates are processed and physician's offices. It is estimated that the reporting is at least 95% complete. Standards of quality-control routines, based on those developed by the International Agency for Research on Cancer^8^ are applied to each tumor record. Every two years a group of specially trained medical students is selected to carry out a field survey of the files of all private physicians who diagnose or treat cancer patients. More detailed methodology has been previously published^5^.

Incidence data were obtained from the Cali Cancer Registry database and mortality data from the Cali Municipal Office of Vital Statistics. The RPCC uses quality assurance procedures, as advocated by IARC, to validate the quality and complementation of cancer registration^8^. Incidence data and quality indices for these data have been published previously in Cancer Incidence in Five Continents (CI5), Volume I-XII^9^ ^-^ ^16^, Table 3. For the period of 2003-2007, the percentage of cases histologically verified was 84.5%, the mortality to incidence ratio (M:I) 49.2% and the percentage of records abstracted from Death certificate only (DCO) 4.5%. Quality Control for Mortality data: 291725 registries were analyzed through 1984-2008, 47,034 (16.1%) corresponding to cancer deaths. A high quality index was observed for cancer death registration with 92.8 % of the cancer deaths properly certified. The percent of missing age at death was 0.2%. The percent of unknown and non-specific primary site of cancer was 6.7%. In almost all cases, the death certificates were signed by a physician (99.7%).

The prevailing International Classification of Diseases (ICD) was used for cancer classification. Three ICD versions have been used through the long registration period. The Eighth Revision, adapted for use in the United States, for 1962 to 1978; the Ninth Revision for 1979 to 1998 and the Tenth Revision for 1998 to 2008. To allow analysis of comparable trends over time, efforts were made to define the cancer sites consistently over the three editions of the ICD. The IARCtools program was used in 1998 to convert codes between ICD versions^17^. The main cancer sites were defined according the Global Burden Diseases 2000 classification of malignant neoplasms by site of primary tumor.^18^ The National Administrative Department of Statistics in Colombia (DANE) provides the distribution of Cali population according to sex, by 5-year age groups. The DANE has organized five censuses of the total Colombian population in 1964, 1973, 1985, 1995 and 2005 ^19^. The age-specific person-years were estimated from these decennial census data with exponential interpolation between censuses. Age-standardized incidence and mortality rates (ASR) were calculated by the direct method, using the world standard population. Rates are expressed per 100,000 person-years^20^ Trends of incidence rates were analyzed during nine quinquennial periods from 1962 to 2007 and trends of mortality rates were analyzed during five quinquennial periods from 1984 to 2008. Trends of rates were evaluated by the annual percentage change (APC), using the weighted least squares method embedded in the US National Cancer Institute's publicly accessible SEER*Stat software^21^ The Annual Percent Change (APC) represents the average percent increase or decrease in cancer rates per year over a specified period of time. In describing the change, the terms "increase" or "decrease" were used when the APC was significantly different from zero (two-sided *p* values <0.05); otherwise the term "stable or flat" was used.

## Results

Table 1 shows the incidence rates per 100,000, for each sex, adjusted to the world population, for the main cancer sites for 9 quinquennial periods from 1962 to 2007. It also includes the trend over time (APC) and its 95% confidence intervals. Table 2 shows mortality rates for the main cancer sites in males and females for 5 quinquennial periods from 1984 to 2008.

**Table 1:**
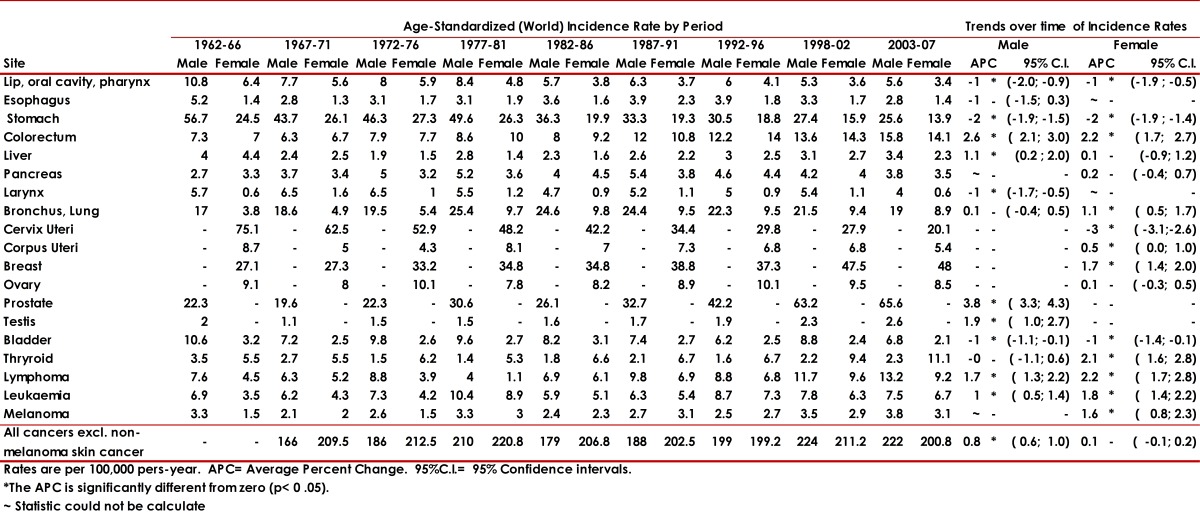
Cali, Colombia. Trends in Age-Standardized (World) Incidence Rates for Selected Sites by sex, through 1962-2007. Rates are per 100,000 pers-years. ~ Statistic could not be calculated. * The APC is significantly different from zero (p <0.05).

### Time trends by cancer site

### Oral cavity and pharynx:

**Figure 2.**
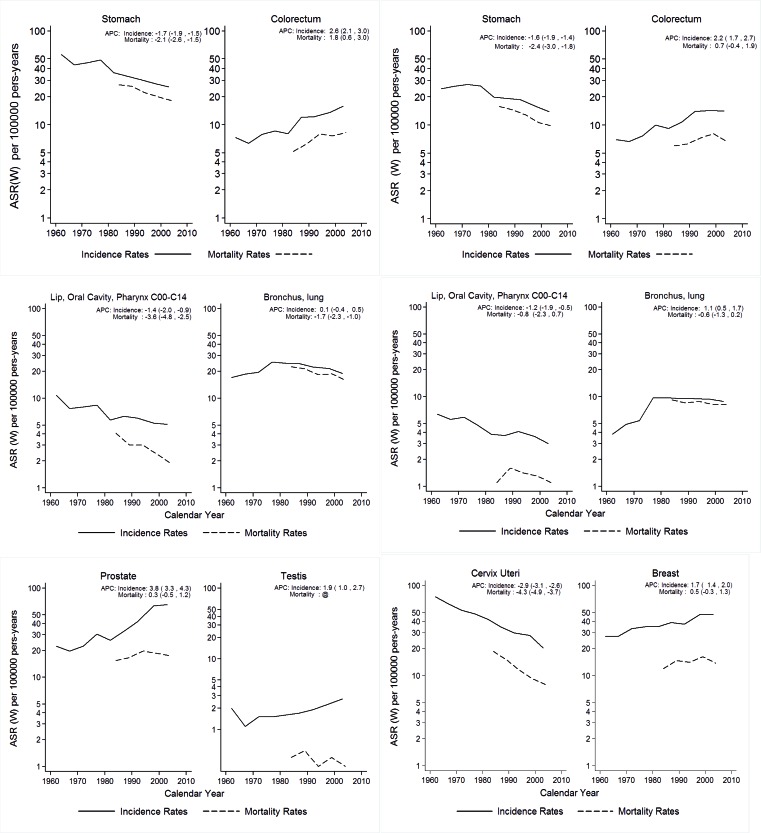
Cali, Colombia. Trends for cancer incidence rates (1926-2007) and cancer mortality rates (1984-2008).

In males incidence rates are higher than in females, and have decreased during the period of observation. The same tendency is seen for females. Mortality rates decreased in men and women (Fig. 2).

### Esophagus:

**Figure 3:**
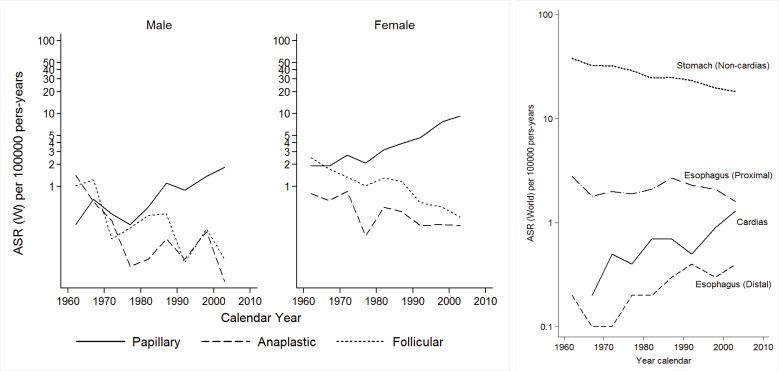
A. Cali, Colombia. Thyroid cancer incidence trends by sex and morphology type of the tumor through 1962-2007. B. Cali, Colombia. Changing the patterns of gastro esophageal junction cancer, 1962-2007.

Incidence rates for proximal esophageal cancer are low, of similar magnitude in both sexes, and have remained stable during the period of observation. Mortality rates have decreased, more markedly in males. Distal esophageal carcinoma has increased since 1970 (Fig. 3B).

### Stomach:

A marked decrease in incidence and mortality rates is observed in both sexes, more evident after around 1980. The rates are higher in men than in women, p<0.05. The incidence rates for carcinoma of the cardia have increased considerably over time (Fig. 3B).

### Colo-rectal cancer:

Incidence and mortality rates are of similar magnitude in both sexes. A steady increase in incidence and mortality rates is observed for both sexes. The increase is observed in all age groups in males. In females the increase is noted only after 50 years of age. Incidence rates in both sexes are steeper after around 1980. The distance between the incidence and mortality rates increases chronologically in both sexes, especially after around 1992.

### Pancreas:

Incidence and mortality rates are rather low in both sexes. Mortality rates show a significant decrease in both sexes (Table 2). 

**Table 2.**
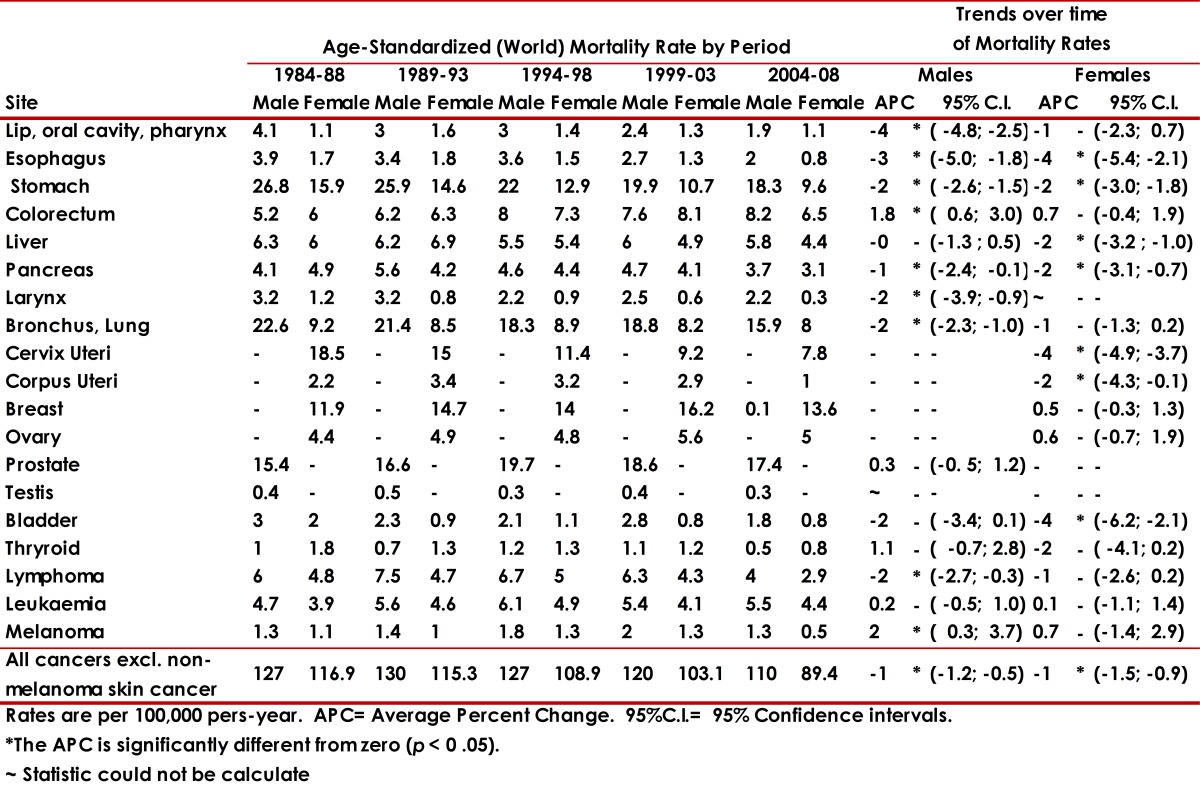
Cali, Colombia. Trends in Age-Standardized (World) Mortality Rates for Selected Sites by sex, through 1984-2008. Rates are per 100,000 pers-years. ~ Statistic could not be calculated. * The APC is significantly different from zero (p <0.05).

### Larynx:

Although the rates are low, a tendency to increase in incidence rates from 1962 to 1976 in males is noted, followed by a slight decrease afterward. Incidence and mortality rates have decreased significantly overall in males. In females the rates are flat.

### Lung cancer:

Incidence and mortality rates are higher in males, *p *<0.05. From 1962 to 1972 incidence rates in both sexes were increasing very slightly. From 1972 to 1981 a sharp tendency to increase was observed in both sexes. After 1981 the rates ceased to increase in both sexes, remaining flat in women and decreasing slightly since 1991 in men. Mortality rates, available only after 1982 are flat until 1991 and show a slight decrease in both sexes. Overall a decreasing trend is noted in both sexes.

### Breast cancer:

The incidence rates increase steadily, more markedly after 1996. The morality rates increase slightly from 1982 to 2005. After 1992 the distance between incidence and mortality rates increases slightly. Age-specific incidence rates show increasing trend in both pre- and post- menopausal cancer.

### Uterine cervix cancer:

A marked and steady and significant decline in incidence and mortality rates is observed during the period of observation. 

### Uterine corpus cancer:

Incidence and mortality rates are low over the time of observation. A modest increase in incidence and decrease in mortality is observed. 

### Ovarian cancer:

Incidence and mortality rates are mostly flat, with small fluctuations. 

### Prostatic cancer:

Incidence rates increased markedly from 1982 to 2005. Mortality rates have remained stable. 

### Testis cancer:

Incidence rates show a significant increase over time. APC for mortality cannot be calculated because of zero values in some years, and mortality rates are low and flat. 

### Bladder cancer:

Incidence and mortality rates are low, lower in females and show decreasing trends in both sexes. 

### Thyroid cancer:

Incidence and mortality rates differ significantly by histologic type. Papillary carcinomas have increased, especially in women. Follicular and anaplastic carcinomas have declined over time (Fig. 3B).

### Melanoma:

In both sexes, mortality and incidence rates are low and have increased with time. 

### Lymphomas:

The incidence has increased with time while mortality rates have decreased in both sexes. In males a slight increase is noticed after around the year 2000. Leukemia incidence rates are low and have increased slightly for both sexes during the period of observation. A small transient increase was registered in both sexes around 1079-1980, coinciding with a "mirror" decrease in lymphomas, apparently reflecting changes in classification rules.

 (Figure 3B) compares cancer incidence rates for the main sites among several Latin American cancer registries. Similar trends are observed for the main cancer sites.

## Discussion

The data presented here mostly confirm and extend trends noticed in a previous publication^22^. They may reflect many interesting facts and trends taking place at the present time in many Latin American countries. In general the population structure has been changing from an African (developing) pattern to a European (developed) pattern. In many cities the immigration from rural areas to large cities has been decreasing, associated with gradual improvement of the socioeconomic conditions. Dietary changes are also taking place associated with urbanization. These demographic and environmental changes are reflected in cancer incidence and mortality patterns, well represented in the data here presented.

It is pertinent to emphasize the major benefits brought to the community by public health campaigns, stimulated and well reflected in the data of the Population-based Cancer Registry. Some of the most remarkable campaigns are the generalized use of vaginal cytology with adequate quality control of laboratory procedures and the national anti-smoking program.

RPCC has documented a steady decrease in incidence rates of gastric cancer after around 1970-1980, not seen in earlier data^22^. The same time trends have been reported in many other countries. Although the exact causes of such trend are not entirely clear, it has been generally linked to the gradual decrease in prevalence rates of *Helicobacter pylori* infection and the steady improvement of living conditions, including diet and home sanitation. In Colombia, the highest incidence rates of gastric cancer are seen in the Andes Mountains, especially in the southern state of Nariño. A positive correlation between altitude over sea level and gastric cancer mortality rates has been reported in Colombia^23^.

**Table 3:**
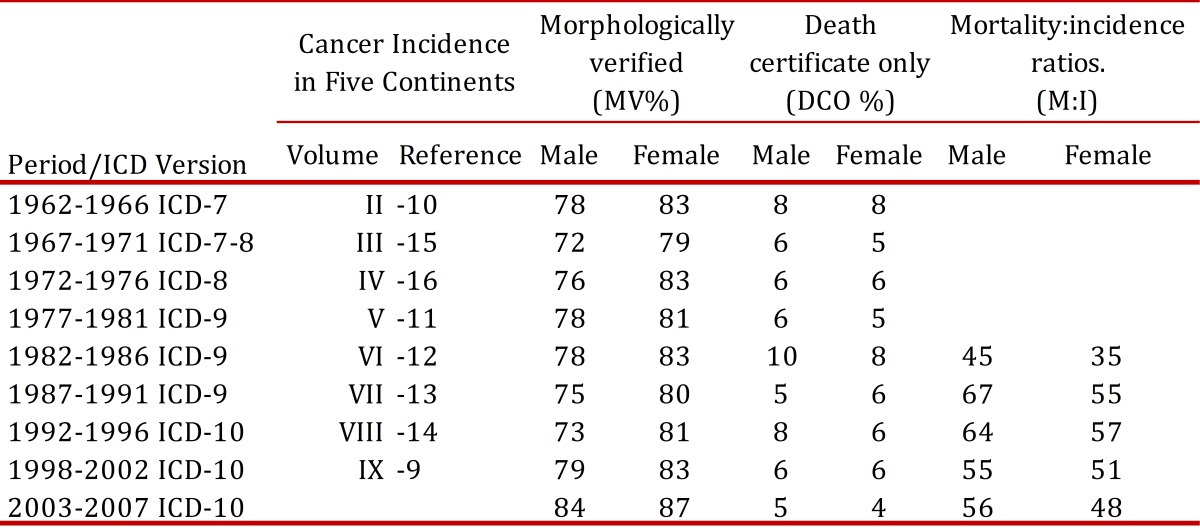
Population Based Cancer Registry of Cali. Data quality and Indices of Reliability trough 1962-2007

The highest rates in Cali are seen in immigrants from Nariño^7^ It has been reported that in Hawaii, the highest rates are seen in immigrants from Japan, but their Hawaii-born descendents have much lower rates^24^. The immigrants to Cali themselves have high prevalence of *H. pylori* infection but their descendants have very low infection rates. The prevalence rates of *H. pylori* infection are very high in Nariño but much lower in the inhabitant of the city of Cali^25^ ^.^ It seems reasonable to assume that the chronologic decrease of gastric cancer rates in Cali is linked to lower *H. pylori* infection prevalence rates and improvements in living conditions. It is also probable that the residents of Cali, including the immigrants, have different dietary patterns that those seen in the rural Andes Mountains, including lower salt consumption and higher intake of fresh fruits and vegetables, known to be associates with lower cancer rates. The trends of colorectal cancer incidence and mortality in Cali show a constant steady increase in the 50 years of observation, first reported in 1983^22^ Age-specific rates for males and females are practically identical. This pattern may reflect the gradual improvement of the socio-economic status of the population. The distance between incidence and mortality rates has been increasing from1982 to 1998, suggesting detection at earlier stages in recent years. In general there is a positive correlation between colorectal cancer and socioeconomic status. Low residue and high fat diet have been implicated.

The causal association between lung cancer and tobacco smoking has been well documented worldwide. Lung cancer incidence rates for both sexes in Cali can be interpreted as reflecting the aborting of a smoking related epidemic that started around 1970 but was interrupted around 1980. It seems safe to conclude that this phenomenon is related to a very strong anti-smoking campaign that has taken place in Colombia since around the 1970's. Mortality rates, available since 1982, never reached epidemic proportions and show a slight decline since around 1992. That campaign seems to have avoided a major health burden for the Colombian population. A similar pattern has been reported in other Latin American countries, such as Ecuador and Venezuela^26^ and has been linked to anti-smoking campaigns^27^.

Breast cancer incidence rates, both pre- and post-menopausal show a steady increase since 1964 which became accentuated around 1992. The same trend has been reported in other Latin American countries, contrasting with decreasing trends in U.S.A and Canada^26^. Mortality rates show a very modest increase between 1982 and 1998; after that date no further increase is observed. As in the case of colon cancer, the general trend may reflect improvements in the socioeconomic status of the population and perhaps some dietary changes. The sudden increase in incidence after 1992, in the absence of a similar trend in mortality rates, may reflect improvements in surveillance and early diagnosis techniques leading to earlier stages at diagnosis, when breast cancer is more curable.

The marked decline in incidence rates for cervical cancer since 1962 most probably reflect the positive effects of screening and prevention programs based on vaginal cytology, as previously documented, accompanied by a marked increase in the relative frequency of carcinoma in situ^22^. Mortality rates have also been declining since 1982.

Incidence rates for prostatic cancer show a marked increase since around 1982, not accompanied by a similar trend in mortality rates. This most probably reflects early diagnosis via PSA testing. This phenomenon has also been observed in many other countries. There is an ongoing discussion about the possible benefits of generalized PSA testing. Many "early" prostatic cancers may never progress to a threatening disease^28^ ^,^ ^29^

The increase in incidence rates of papillary carcinoma of the thyroid gland in women was noticed in an earlier publication^22^ ^.^ No clear causal association is available. It seems to coincide with trends for breast and colon carcinomas. The observed decrease in incidence of follicular carcinoma was predicted previously^22^ and may reflect the end of the epidemic of endemic goiter brought about by iodine addition to table salt^4^.

During its 50 years of continuous operation the RPCC has had a clear impact on the national and the Latin American field of health services. It has fostered and guided the establishment of other Colombian population-based registries in the cities of Pasto, Manizales, Bucaramanga and Barranquilla.

The Registry has provided accurate and relevant information for the education of health professionals and supported community efforts to design and establish programs for the prevention and control of cancer. Internationally, it has contributed to all issues of the International Agency for Research on Cancer (IARC) on cancer statistics: Cancer Incidence in Five Continents and GLOBOCAN.

It inspired the creation and guided the operation of the Population-based Cancer Registry of Quito, Ecuador, now in continuous operation for 19 years. The new Latin American Registries will be valuable in comparative studies of the incidence of specific types of tumors in populations with considerable demographic and geographic diversity.
